# Revisiting the role of hypoxia-inducible factors and nuclear factor erythroid 2-related factor 2 in regulating macrophage inflammation and metabolism

**DOI:** 10.3389/fcimb.2024.1403915

**Published:** 2024-07-25

**Authors:** Kenneth K. Y. Ting

**Affiliations:** ^1^ Department of Immunology, University of Toronto, Toronto, ON, Canada; ^2^ Toronto General Hospital Research Institute, University Health Network, Toronto, ON, Canada

**Keywords:** HIF-1a hypoxia-inducible factor-1a, NRF2, macrophage, LPS, inflammation, NADPH, immunometabolism, redox

## Abstract

The recent birth of the immunometabolism field has comprehensively demonstrated how the rewiring of intracellular metabolism is critical for supporting the effector functions of many immune cell types, such as myeloid cells. Among all, the transcriptional regulation mediated by Hypoxia-Inducible Factors (HIFs) and Nuclear factor erythroid 2-related factor 2 (NRF2) have been consistently shown to play critical roles in regulating the glycolytic metabolism, redox homeostasis and inflammatory responses of macrophages (Mφs). Although both of these transcription factors were first discovered back in the 1990s, new advances in understanding their function and regulations have been continuously made in the context of immunometabolism. Therefore, this review attempts to summarize the traditionally and newly identified functions of these transcription factors, including their roles in orchestrating the key events that take place during glycolytic reprogramming in activated myeloid cells, as well as their roles in mediating Mφ inflammatory responses in various bacterial infection models.

## Introduction

Immunometabolism is an emerging field of research that studies the metabolic regulation of immune function by integrating both biochemistry and immunology on a cellular and systemic level. Although the term “immunometabolism” first appeared in literature in 2011, the importance of metabolites for immune cell function has been reported many years ago (Alonso and Nungester, 1956; Oren et al., 1963; Newsholme et al., 1986; Fukuzumi et al., 1996; Mathis and Shoelson, 2011). In fact, intracellular metabolism has always been recognized to play a role more than bioenergetics. For instance, the difference in amino acid metabolism has been traditionally used to define macrophages (Mφs) subsets, with M1 or M[LPS(+IFNγ)] converting arginine into nitrogen oxide (NO) via inducible Nitric Oxide Synthase (iNOS), while M2 or M[IL-4] converting arginine into ornithine via arginase-1 (Corraliza et al., 1995; Modolell et al., 1995; Munder et al., 1998; Murray et al., 2014). Interestingly, in the past, intracellular metabolism was not included in immunological research except for the studies of metabolic diseases, such as obesity or diabetes (O'Neill et al., 2016). This was attributed to a lack of robust and sensitive technologies to directly assess how metabolic pathways are linked to immune cell functions. However, recent advancements made in technologies, such as mass spectrometry-based metabolomics and Seahorse analyzers, now confer possible means to study the contribution of metabolic changes in immune functions.

Among all the immune cell types, the metabolic functions of myeloid cells, such as Mφs and Dendritic cells (DCs), are the most characterized. First discovered by Metchnikoff for their abilities to phagocytose and kill microbes, Mφs are now appreciated as innate immune cells that can detect infection or tissue damage by expressing pattern recognition receptors (PRRs), which are germline-encoded receptors that can bind to conserved compounds derived from microbes or damaged tissues (Medzhitov and Janeway, 2000; Seong and Matzinger, 2004). The successful binding of these ligands to their receptors then activate a series of phosphorylation cascades, which relay this sensing information intracellularly and activate the corresponding transcription factor to transcribe the appropriate set of genes in response to the external stimuli. The classical example of this is the binding of lipopolysaccharide (LPS) to Toll-like receptor 4 (TLR4). LPS is a molecule derived from the outer membranes of gram-negative bacteria, therefore, it is often used as a mimetic of bacterial infection. Upon binding to TLR4, LPS activates a series of phosphorylation cascades, such as Akt and MAPKs, then the subsequent activation of transcription factors, such as NF-кB and AP-1, leading to the transcription of genes related to inflammation, phagocytosis and killing of bacteria. Although the signaling and transcription events post LPS-stimulation has been well characterized, how intracellular metabolic circuits support these events remained a mystery until the metabolites involved were profiled.

In 2010, it was first described in Mφs that LPS stimulation led to the enhancement of glycolytic flux, which can be divided into two time-dependent phases: 0-4hr and 4-12hr (Rodríguez-Prados et al., 2010). The first phase of enhancement, known as the early stage of glycolytic reprogramming, was much slower than the second phase, known as the late stage of glycolytic reprogramming. Transcriptional analyses revealed that during the enhancement of glycolytic flux, the expression of genes related to glycolysis was upregulated while the genes that encode for proteins involved in the oxidative phosphorylation were suppressed. Similar findings were reported in activated DCs whereby TLR agonists induced a metabolic switch to glycolytic metabolism with an impairment in mitochondrial respiration (Krawczyk et al., 2010; Everts et al., 2012). Taken together, these studies have demonstrated that LPS-activated myeloid cells adopt a unique metabolic profile analogous to the Warburg effect, which is a form of cellular metabolism in tumor cells characterized with high levels of glucose uptake, conversion from glucose to lactose, and a decline in oxidative phosphorylation levels (Warburg et al., 1927).

Subsequent studies published after the aforementioned ones have now elucidated the molecular mechanisms behind the induction of glycolysis and its linkage with inflammation, as well as the induction of the resolution phase of inflammation. Notably, the transcriptional regulation mediated by Hypoxia-Inducible Factors (HIFs) and Nuclear factor erythroid 2-related factor 2 (NRF2) orchestrate some of these key mechanisms. Thus, their traditionally well-characterized roles and newly elucidated functions are summarized in this review, followed by a revisit of the key events that take place during the glycolytic reprogramming in myeloid cells.

## Hypoxia-inducible factors

Hypoxia-Inducible Factors (HIFs) is a family of transcription factors that are known to activate the transcription of genes in response to decrease in available oxygen, or hypoxia. Although HIFs were first discovered in 1991 for their role in mediating cellular responses against hypoxia (Semenza et al., 1991), it is now revealed that they also plays a vital role in mediating the transcriptional programs related to inflammation, angiogenesis, survival, and glucose metabolism (Ruas and Poellinger, 2005; Tannahill et al., 2013). The importance of discovering this family of transcription factors was eventually recognized by awarding the 2019 Nobel Prize in Physiology or Medicine to Gregg Semenza, Peter Ratcliffe and William Kaelin, the three scientists that significantly contributed to our understanding of how cells sense and adapt to oxygen availabilities.

**Figure 1:**
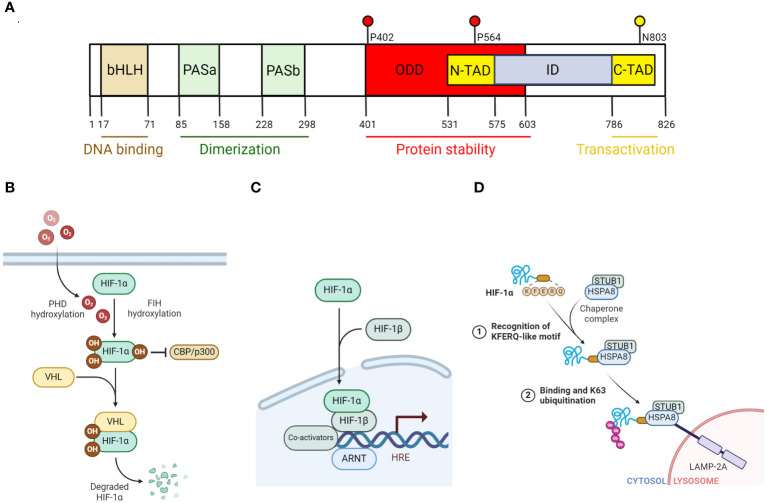
The regulation of HIF-1α degradation and function. **(A)** Diagram that illustrates the different domains of HIF-1α. bHLH and PAS domains are important for the formation of heterodimeric complex with HIF-1β and binding to DNA. N-TAD domain contains key proline residues (P402, P564) where prolyl hydroxylase (PHD) hydroxylates. C-TAD domain contains a key asparagine residue (N803) that factor inhibiting HIF (FIH) hydroxylates. **(B)** Diagram that illustrates PHD-VHL-dependent degradation of HIF-1α and the inhibition of HIF-1α transactivation capacity by FIH under normoxic condition. **(C)** Diagram that illustrates HIF-1 transcriptional activation of its targeted genes. Specifically, HIF-1α that escaped from its proteasomal degradation, combines with HIF-1β, to form a heterodimer transcription factor known as HIF-1. Along with its co-activators (such as PKM2, p300/CBP), HIF-1 then binds to its targeted genes, which are characterized with a hypoxia response element (HRE) and transcribes them. **(D)** Diagram that illustrates HIF-1α degradation via the non-canonical chaperone-mediated autophagy pathway. In this model, HSPA8 recognizes the KFERQ-like motif of HIF-1α and binds to it. STUB1, which is associated with HSPA8 as a complex, catalyzes K63 ubiquitination on HIF-1α, targeting it for lysosomal degradation in a LAMP2A-dependent manner. All figures are made by Biorender.com.

In general, members of the HIF family are heterodimeric beta helix-loop-helix (HLH) transcription factors composed of an α and a β subunit, in which the α subunit is unstable and oxygen sensitive, while the β subunit is constitutively expressed and insensitive to oxygen. To date, three known α subunits, HIF-1α, HIF-2α and HIF-3α, as well as three β subunits, HIF-1β, HIF-2β and HIF-3β, have been identified (Reisz-Porszasz et al., 1994; Wang and Semenza, 1995; Jiang et al., 1996; Jiang et al., 1997; Pugh et al., 1997; Huang et al., 1998; Ema et al., 1999; O'Rourke et al., 1999; Masson et al., 2001). Among all, HIF-1α and HIF-2α are the most characterized. For HIF-1α specifically, it is structurally characterized with a N-terminal basic HLH followed by Per-ARNT-Sim (PAS) domains, oxygen-dependent-degradation (ODD) domains, and two transcription activation domains in the C-terminus (**Figure 1A**). The N-terminal basic domain is critical for HIF-1 binding to the consensus sequence of its target genes, which is known as hypoxia-response elements (HRE): 5’-TACGTG-3’ (Wang and Semenza, 1993; Melillo et al., 1995). The HLH and PAS domains on the other hand are important for the heterodimerization between the α and β subunits. The ODD domains contain critical conserved proline residues, where they can be hydroxylated by prolyl hydroxylases (PHDs) in the presence of oxygen and target HIF-1α for proteasomal degradation (Ivan et al., 2001; Jaakkola et al., 2001; Masson et al., 2001; Yu et al., 2001). Finally, there are two transcription activation domains in the C-terminus: N terminal transactivation domain (N-TAD) and C terminal transactivation domain (C-TAD). While N-TAD is located within the ODD domain, C-TAD is near the end of the C-terminus and harbors a conserved asparagine residue, in which its hydroxylation can inhibit the transactivation capacity of HIF-1α (Mahon et al., 2001).

## The regulation of HIF-1α stability

Given the short half-life of HIF-1α (~5 minutes) (Huang et al., 1998), past efforts in research have been dedicated to elucidating the mechanisms behind the regulation of HIF-1α degradation. In general, HIF-1α can be degraded by the classical ubiquitin proteasome system (UPS), which is dependent on proline hydroxylases (PHDs) and von Hippel-Lindau (VHL) ubiquitin ligase, or alternatively via the autophagy-lysosomal pathway (Hubbi et al., 2013). Other non-canonical pathways that mediate HIF-1α degradation independent of UPS and autophagy have also been reported in past studies (Iommarini et al., 2017). In the UPS model, both oxygen-dependent proline hydroxylation by PHDs and VHL-mediated ubiquitination are required (Maxwell et al., 1999; Cockman et al., 2000; Ohh et al., 2000; Bruick and McKnight, 2001; Epstein et al., 2001). Specifically, under normoxic condition, PHDs hydroxylate HIF-1α on two proline residues, Pro402 and Pro564 (for human HIF-1α), within the ODD domain (Ivan et al., 2001; Jaakkola et al., 2001; Masson et al., 2001; Yu et al., 2001). The hydroxylation sites allow VHL to mediate ubiquitination and eventually proteasomal degradation (**Figure 1B**). However, under hypoxic condition, without oxygen, PHDs activity is inhibited and thus prevent HIF-1α from being degraded. The stabilized HIF-1α can then translocate into the nucleus where it forms a dimer with HIF-1β, which constitutes the HIF-1 complex required for the transcription of its targeted genes (**Figure 1C**).

As previously mentioned, HIF-1α can also be degraded by the autophagy-lysosomal pathway under certain conditions, specifically, by macroautophagy or chaperone-mediated autophagy (CMA). Macroautophagy involves the sequestration of long-lived proteins and aggregates within the autophagosome and delivering it to the lysosome for degradation. This process is mediated by Sequestosome 1 (SQSTM1) or neighbor of BRCA1 gene 1 (NBR1) (DePavia et al., 2016). Indeed, past studies have shown blocking autophagic inhibition by knocking down SQSTM1 impaired the induction of HIF-1α by Q6, a hypoxia-activated prodrug (Liu et al., 2014). This notion was reinforced by the reduction of HIF-1α levels upon AZD-2014-mediated inhibition of mammalian target of rapamycin complex 1/2 (mTORC1/2), which activates autophagy (Zheng et al., 2015). Unlike macroautophagy, CMA involves heat-shock cognate protein of 70kDA (HSC70) targeting its substrate, such as HIF-1α (Hubbi et al., 2013), to lysosome-associated membrane protein type 2A (LMAP2A) for degradation. Under this specific condition, E3 ligase STIP1 homology and U-box containing protein 1 mediate HIF-1α K63 ubiquitination instead of VHL (Ferreira et al., 2013; Ferreira et al., 2015) (**Figure 1D**).

Finally, past studies have reported that under specific conditions, HIF-1α can also be degraded without the involvement of VHL and lysosomes. Specifically, in these studies, authors have shown that WD repeat and SOCS box-containing protein 1 (WSB1) can stabilize HIF-1α by ubiquitinating VHL in cancer cells (Kim et al., 2015). MDM2, another E3-ubqiutin-protein ligase, was also reported to ubiquitinate HIF-1α independent of its hydroxylation by binding to tumor suppressor proteins, such as TAp73 or p53 (Ravi et al., 2000; Amelio et al., 2015). Past studies have also shown that post-translational modifications other than hydroxylation, such as SET7/9-mediated methylation, LSD1-mediated demethylation, p300-mediated acetylation, SIRT2-mediated deacetylation, can all regulate HIF-1α stability (Geng et al., 2012; Seo et al., 2015; Kim et al., 2016). Taken together, these studies have revealed the diverse ways of how HIF-1α stability can be regulated, and such diversity may allow the fine-tuning of HIF-1 transcriptional output in response to a wide range of external stimuli.

## The regulation of HIF-1α transactivation capacity

Apart from the proline hydroxylation mediated by PHDs, HIF-1α is also hydroxylated by Factor Inhibiting HIF (FIH) at Asn803 (human HIF-1α) located within the C-TAD domain during normoxic condition (Mahon et al., 2001). This asparagine hydroxylation inhibits the transcriptional activity of HIF-1 by sterically blocking the recruitment of coactivators CBP/p300 (Mahon et al., 2001). Similar to PHDs, FIH catalyzes its hydroxylation reaction via the oxidative decarboxylation of 2-oxoglutarate, producing carbon dioxide and succinate as by products. Like PHDs, the enzymatic activity of FIH is also critically dependent on its catalytic center of iron in its ferrous state (Fe^+2^), which is maintained by the reducing action of ascorbic acid (Knowles et al., 2003; Pagé et al., 2008). Knockout studies of FIH revealed that it is an essential regulator of metabolism as mice deficient of FIH have decreased body masses with an elevated metabolic rate (Zhang et al., 2010). Moreover, these mice also have improved glucose and lipid homeostasis, thus making them resistant to weight gain induced by high-fat-diet (Zhang et al., 2010).

Although FIH and PHDs are iron (II)-dependent dioxygenases, they have significant structural differences, which underlie their differential regulation of HIF-1 transcriptional output. For instance, FIH has broad functional pockets that allow O_2_ and other co-factors to bind tightly, whereas PHDs have a narrow opening of active site (Lee et al., 2003; Flashman et al., 2010). This structural difference enables FIH to work efficiently despite low O_2_ availability, while PHDs remain inactive in these situations (Rani et al., 2022). More importantly, this difference in the binding affinity for O2 between FIH and PHDs underlies the graded HIF-1 transcriptional response in response to a wide range of hypoxic conditions. Apart from their differences in binding affinity for O2, FIH and PHDs also exhibit differences in their functional inhibition by TCA cycle metabolites and oxidative stress. For instance, an *in vitro* study has shown that while succinate and fumarate can competitively inhibit PHDs by occupying their active sites, they have no effect on FIH (Koivunen et al., 2007). On the other hand, citrate and oxaloacetate can inhibit both PHDs and FIH (Koivunen et al., 2007). In response to oxidative stress, FIH was found to be more sensitive to its inhibition in comparison to PHDs. Specifically, one *in vitro* study has shown that the activity of FIH was significantly more inhibited by tert-butyl hydroperoxide-induced oxidative stress than PHDs (Masson et al., 2012). Taken together, these studies demonstrate that the differential responses between FIH and PHDs to external stimuli underlie the spectrum of HIF-1 transcriptional reprogramming.

Finally, apart from catalyzing asparagine hydroxylation, an early *in vitro* study has also revealed that FIH could perform secondary functions, such as acting as a corepressor with VHL to block histone deacetylases from binding to DNA (Mahon et al., 2001). Indeed, another recent study has shown that overexpressing FIH can inhibit the mRNA of GLUT1 even in hypoxia condition, in addition to normoxic condition (Wang et al., 2014). This reinforces the possibility that FIH can regulate HIF-1 transcriptional activity independent of its hydroxylation activity.

## The inflammatory role of HIF-1α in LPS-activated Mφs

Although the transcriptional role of HIF-1α under hypoxic condition is well established, its role in mediating inflammation in activated Mφs has only been recently elucidated. The investigation first began in 2003 with the initial characterization of myeloid cells that are genetically deficient of *Hif1a* from *Ly2Cre-Hif1a^fl/fl^* mice (Cramer et al., 2003). In this study, the authors found that myeloid cells that lack HIF-1α have impaired ATP levels, motility, invasiveness, bactericidal activity, and aggregation (Cramer et al., 2003), thereby implicating the role that HIF-1α plays in regulating metabolism and inflammation. This notion was further supported later by studies that show overexpressing HIF-1α leads to the upregulation of M1 signature markers (Takeda et al., 2010; Wang et al., 2017) post LPS and IFNγ stimulation. Taken together, these earlier studies have illustrated a positive role that HIF-1α plays in mediating an inflammatory response in activated M1 Mφs.

To elucidate the mechanisms underlying HIF-1α involvement in inflammatory response in activated Mφs, chromatin immunoprecipitation of HIF-1α was performed and revealed that HIF-1α binds to many glycolytic genes, such as *Slc2a1, Ldha, Hk2, Pfkp*, as well as proinflammatory genes, such as *Il1b* and *Nos2* in LPS-activated Mφs (Melillo et al., 1995; Peyssonnaux et al., 2007; Tannahill et al., 2013). The transcription of glycolysis genes is critical for driving Mφs towards an aerobic glycolytic metabolism, which is essential for fueling many intracellular inflammatory machineries, such as Pentose Phosphate Pathway (PPP)-derived NADPH production for Nitric Oxide Synthase (NOSes) and NADPH Oxidases (NOXes), TCA cycle-derived citrate for *de novo* lipid synthesis. On the other hand, the transcription of proinflammatory genes directly contributes to the production of inflammatory cytokines. Apart from LPS stimulation, RNA-sequencing analysis also revealed that almost half of Interferon gamma (IFNγ)-induced genes in Mφs are HIF-1α-dependent (Braverman et al., 2016). Taken together, these studies have revealed the direct and indirect roles that HIF-1α play in orchestrating an effective inflammatory response in an activated Mφs.

Understanding how important the transcriptional role of HIF-1α is in activated Mφs, there is now an increasingly growing interest to elucidate the molecular factors that are required for its transcriptional activity. For instance, recent studies have shown that PKM2, which is a pyruvate kinase in the last rate-limiting step in glycolysis, can act as a cofactor for HIF-1α (Luo et al., 2011; Palsson-McDermott et al., 2015) post LPS stimulation. Specifically, PKM2 can transition from its active tetrameric state to its dimeric state, then translocate into the nucleus and interact with HIF-1α to promote its transcriptional regulation. Inhibitors, such as DASA-58 and TEPP-46, that block the dimerization of PKM2 could impair HIF-1 activity and reduced inflammation in *in vivo* pathogenic models (Palsson-McDermott et al., 2015). Overall, these studies have identified the cofactors needed for HIF-1 transcription, and how the manipulation of these cofactors confer possible means to regulate HIF-1 transcriptional output for therapeutic purposes.

## Introduction to nuclear factor erythroid 2-related factor 2

Reactive oxygen species (ROS), i.e. super oxide anions (O_2_^•−^) and hydrogen peroxide (H_2_O_2_), and reactive nitrogen species (RNS), i.e. nitric oxide (NO)-derived peroxynitrite (ONOO^-^), are generated as a result of intracellular metabolism and upon exposure to extracellular stimuli (Ma, 2010; Finkel, 2011). Although their uncontrolled production can result in oxidative stress that abrogates cellular function and contributes to the development of many inflammatory diseases, when regulated properly, oxidants are important signaling molecules for a wide range of cellular processes (Finkel, 2011; Sies et al., 2022), as well as mediating the inactivation of pathogens. For instance, the generation of O_2_^•−^ from NOX2 is critical for the anti-microbial function of Mφs (Brüne et al., 2013). Similarly, the generation of mitochondrially-derived ROS has been recently shown to be activated by TLR4 signaling and critical for Mφ anti-bacterial responses (West et al., 2011). Finally, the generation of ROS from other sources, including xanthine oxidase (Ives et al., 2015) and peroxisomes (Di Cara et al., 2017) have also been reported to play an important role in mediating the intracellular killing of bacteria in Mφs.

A key transcription factor that regulates the abundance of ROS is known as the nuclear factor erythroid 2 (NFE2)-related factor 2 (NRF2). Similar to Nrf1 and Nrf3, NRF2 belongs to the cap “n” collar (CNC) subfamily of basic-region leucine zipper (bZIP) transcription factor (Oyake et al., 1996). First discovered in 1994 for its role in erythropoiesis and platelet development (Moi et al., 1994), it was later revealed that it was essential for mediating the induction of drug-metabolizing and ROS detoxification enzymes, such as NAD(P)H:quinone oxidoreductase 1 (Itoh et al., 1997). All these enzymes share a common DNA sequence, known as the antioxidant response element (ARE), where NRF2 binds to (Nguyen et al., 2003). Specifically, the ARE sequence is 41-base-pair, and contains a conserved sequence, 5’-TGACnnnGC-3’, where n represents any base (Rushmore et al., 1991).

**Figure 2:**
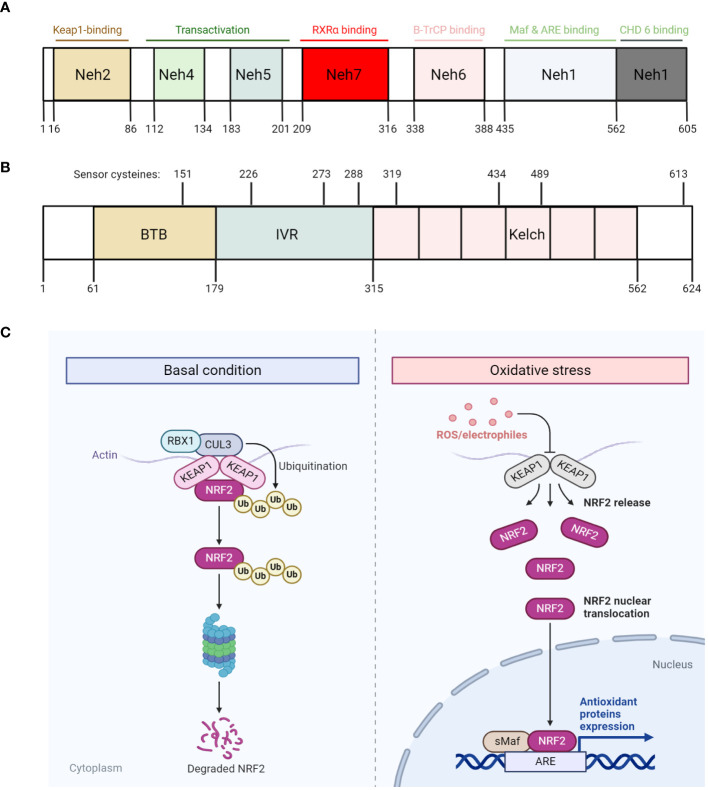
The regulation of NRF2 degradation and function. **(A, B)** Diagram that illustrates the different domains of NRF2 **(A)** and KEAP1 **(B)**, with each domain harbors different functions. Specifically, KEAP1 also harbors various critical cysteine residues that readily respond to oxidative stress. **(C)** Diagram that illustrates NRF2 degradation and function under basal condition (left) and oxidative stress (right) respectively. Under basal condition, NRF2 is constitutively degraded by KEAP1 through the proteasome. However, under oxidative stress condition, ROS/electrophiles disrupt KEAP1 conformation by reacting with its critical cysteine residues, thus releasing NRF2 and allowing it to escape from degradation. The escaped NRF2 then translocate into the nucleus, along with sMaf proteins, bind to its targeted genes, which are characterized with antioxidant response element (ARE), and transcribes them. All figures are made by Biorender.com.

Given the importance of NRF2 in regulating cellular responses to oxidative stress, past decades of research have been invested in elucidating the regulation of NRF2 function and found that NRF2 is always degraded under basal conditions by Kelch-like erythroid cell-derived protein with CNC homology-associated protein 1 (KEAP1)-mediated ubiquitination-proteasomal degradation. KEAP1 was identified as a substrate adaptor of CUL3 (a E3 ubiquitin ligase) via a yeast two-hybrid screen (Itoh et al., 1999). The molecular and structural analysis of NRF2 has revealed that it contains seven NRF2-ECH homology domains (Neh1-7), with each domain serving a distinct function (Hayes and Dinkova-Kostova, 2014) (**Figure 2A**). For instance, the Neh1 domain includes a CNC-bZIP region that is critical for the DNA binding activity of NRF2, as well as its association with small musculoaponeurotic fibrosarcoma (sMaf) proteins as dimerization partners (Motohashi and Yamamoto, 2004). The Neh2 domain contains the highly conserved DLG and ETGE motifs, which are important for the interaction between KEAP1 and NRF2. This domain also includes the seven lysine residues where they can be ubiquitylated and mediate NRF2 for subsequent proteasomal degradation (Itoh et al., 1999; McMahon et al., 2003; Tong et al., 2006). The Neh3 domain, which contains the transactivation activity, activates the transcription of NRF2 targeted genes, together with Neh4 and Neh5 domains (Katoh et al., 2001; Nioi et al., 2005; Sekine et al., 2016). Finally, Neh6 domain is involved in regulating NRF2 stability independent of KEAP1 (Chowdhury et al., 2013), while Neh7 domain is involved in suppressing the transcriptional activity of NRF2 (Wang et al., 2013). Overall, these studies have demonstrated the intimate link between the structure of NRF2 and the regulation of its function.

## The regulation of NRF2 stability

The relatively short half-life of NRF2 (18.5 min) suggests its function is primarily regulated by its stability (Itoh et al., 2003). Indeed, cycloheximide-mediated inhibition of protein synthesis impeded basal and induced expression of NRF2-targeted genes, and that NRF2 can be stabilized with its half-life increased up to 200 min (Ma, 2013; Canning et al., 2015). Since NRF2 is a master transcription factor regulating antioxidative defense mechanisms, its basal levels are always maintained to be very low due to its constant ubiquitination and proteasomal-degradation mediated by the KEAP1/CUL3 complex. As previously mentioned, KEAP1 acts as a substrate adaptor by linking NRF2 to CUL3, thereby mediating the ubiquitination of NRF2 by CUL3. Interestingly, the interaction between KEAP1 and NRF2 is always in a ratio of 1:2, where two KEAP1 proteins interact with one NRF2 as a dimer within the Neh2 domain (Padmanabhan et al., 2006; Tong et al., 2006; Lo et al., 2017). Specifically, structural analyses of KEAP1 have shown that KEAP1 contains two protein-interacting domains, known as the BTB domain in the N-terminal region, and the Kelch repeats in the C-terminal region (**Figure 2B**). While the BTB domain mediates the binding of KEAP1 to CUL3, the Kelch repeats mediate the binding of KEAP1 to NRF2 (Li et al., 2004; Padmanabhan et al., 2006; Ogura et al., 2010). In between the Kelch repeats and BTB domain lies the linker region, which is rich in cysteine residues that are prone to be oxidized by reactive radicals or electrophilic reagents. Under conditions where redox homeostasis is disrupted (i.e. ROS overproduction), these radicals can readily react with these key cysteine residues, including C151, C273 and C288 (Eggler et al., 2005; Hong et al., 2005; He and Ma, 2010; McMahon et al., 2010; Baird et al., 2013), and presumably induce a conformational change of KEAP1. This enables NRF2 to dissociate from KEAP1 and translocate to the nucleus where it activates the expression of genes that encode antioxidative proteins, such as catalase, glutathione-disulfide reductase and thioredoxin reductase (**Figure 2C**).

Apart from the classical model of KEAP1/CUL3-mediated NRF2 ubiquitination, past studies have reported that NRF2 stability can be modified by other post-translational modifications, and interactions with other proteins. For instance, NRF2 can be phosphorylated by ERK, JNK, AKT, PKC, CK2 and PERK, in which their phosphorylation increases the stability of NRF2 (Huang et al., 2002; Cullinan et al., 2003; Yuan et al., 2006; Apopa et al., 2008; Hayes et al., 2015). On the other hand, p38 and GSK3-mediated phosphorylation destabilizes NRF2 (Hayes and Dinkova-Kostova, 2014). Apart from phosphorylation, NRF2 was also shown to be glycated, and its deglycation indirectly induced by fructosamine-3-kinase stabilizes NRF2 (Sanghvi et al., 2019).

In recent years, the regulation of NRF2 by SQSTM1 (also known as p62) is increasingly appreciated. First reported in 2007, Liu et al. found that the overexpression of p62 led to NRF2 nuclear translocation and activation of NRF2-targeted genes (Liu Y. et al., 2007). The underlying mechanism was then elucidated three years later by several groups, where they found that p62 interacts with KEAP1, specifically the NRF2-binding site. Therefore, an ectopic expression of p62 can outcompete NRF2 for KEAP1, sequestering KEAP1 into aggregates or autophagosomes and impedes KEAP1-mediated NRF2 degradation (Copple et al., 2010; Fan et al., 2010; Komatsu et al., 2010; Lau et al., 2010). Interestingly, an ARE element was also found to be in the p62 promoter, thereby supporting the notion that a positive feedback loop exists between the transcription of p62 and inhibition of NRF2 degradation (Jain et al., 2010). Apart from this, another positive feedback loop also exists between p62-mediated KEAP1 degradation and activation of NRF2, as reported by Copple et al. In their study, they found that overexpression of p62 significantly accelerated the degradation of KEAP1 whereas the inhibition of p62 expression increased KEAP1 expression (Copple et al., 2010). Similar results were also reported in liver-specific, *p62-*deficient mice, in which the liver-specific expression of KEAP1 was significantly reduced in comparison to its wild-type counterparts (Taguchi et al., 2012). Overall, it is generally believed that the p62-dependent non-canonical pathway results in prolonged activation of NRF2, in which this condition is found to be favorable for cancer cells to thrive as it confers cryoprotection and antioxidative defense mechanisms (Jiang et al., 2015). Taken together, these studies have demonstrated how NRF2 stability can be diversely regulated, in which this diversity is critical to optimize NRF2 transcriptional output in response to a wide range of oxidative stress conditions.

## The anti-inflammatory role of NRF2 in LPS-activated Mφs

Although the role that NRF2 plays in regulating redox homeostasis is well-established, how it regulates intracellular metabolism and how this is linked to antioxidative defense in activated Mφs is not completely understood. Early studies have revealed both redox-dependent and independent roles that NRF2 play in suppressing inflammation in LPS-activated Mφs. For instance, it was discovered that NRF2-activation by itaconate-mediated alkylation of KEAP1 suppressed type I IFN responses in LPS-activated Mφs (Mills et al., 2018). Specifically, authors in this study found that itaconate and 4-octyl itaconate, which is a cell-permeable itaconate derivate, deactivated KEAP1 by alkylation and thereby activated NRF2-dependent antioxidative defense mechanisms (Mills et al., 2018). The upregulation of NRF2 then led to an impaired induction of type I IFN responses, HIF-1α levels, proinflammatory cytokine production and glycolysis (Mills et al., 2018). Overall, the study suggested that NRF2 controls Mφ inflammation by metabolic regulation. In contrast to this study, another study has found that NRF2 could suppress the transcription of LPS-induced *Il1b* and *Il6* by competing with RNA Polymerase II binding (Kobayashi et al., 2016). Specifically, in this study, the authors used ChIP-seq and ChIP-qPCR analyses to demonstrate that in *Ly2Cre: Keap1^fl/fl^* mice where NRF2 is overexpressed in myeloid cells, NRF2 could bind to the proximity of Il1β and Il6, which blocked the recruitment of RNA Polymerase II binding to their transcriptional start sites (Kobayashi et al., 2016). Overall, these studies have shown that NRF2 can regulate inflammation in ROS-dependent and independent ways.

How NRF2 controls inflammation by metabolic regulation remains unclear. Recent studies have begun to characterize the metabolism of Mφs by manipulating levels of NRF2 via genetic and pharmacological means (Ding et al., 2019; Mornata et al., 2020; Ryan et al., 2022). For instance, a recent study has profiled the proteome, metabolome, and transcriptome of bone marrow-derived Mφs (BMDMφs) isolated from WT, KEAP1-knockout or NRF2-deficient mice (Ryan et al., 2022). In this study, the authors have found that NRF2 significantly altered the proteome post LPS stimulation, including alterations in redox, carbohydrate and lipid metabolism (Ryan et al., 2022). Interestingly, the authors also found that NRF2 can modulate mitochondrial morphology, specifically, promoting their morphological transition from intermediate to fused/elongated forms post LPS stimulation (Ryan et al., 2022). Taken together, the authors found a correlation between these metabolic changes to the suppression of IFNβ responses in LPS-activated Mφs. Apart from BMDMφs, recent research has also identified an enrichment of NRF2-mediated transcriptional signature in tumor-induced myeloid-derived suppressor cells (MDSCs), which are immature myeloid cells that inhibit the activation of T cells (Gabrilovich et al., 2007), the cytotoxic functions of NK cells (Liu C. et al., 2007) and the induction of T regulatory cells (Huang et al., 2006). For instance, Beury et al. have first reported that NRF2 activation in MDSCs protects them from oxidative stress and apoptosis, thereby increasing its suppressive abilities and infiltration in tumors (Beury et al., 2016). Metabolically, Ohl et al. have elucidated that NRF2-induction in MDSCs increased their nutrient uptake through glycolysis, PPP and mitochondrial metabolism, thus supporting their proliferative abilities (Ohl et al., 2018). With respect to the pathways that activate NRF2 in MDSCs, recent research performed by Mohamed et al. have found that PKR-like endoplasmic reticulum (ER) kinase (PERK) signaling leads to NRF2-activation and its regulation on mitochondrial respiratory homeostasis, in which is critical for the immunosuppressive functions of MDSCs (Mohamed et al., 2020). Indeed, the authors found that the suppression of PERK expression abrogated NRF2 signaling in MDSCs, increased mitochondrial DNA content and activated STING-dependent expression of anti-tumor Type I IFN responses. Overall, these studies have demonstrated that NRF2 can exert its anti-inflammatory effects by intrinsically rewiring the metabolism of activated inflammatory immune cells or by supporting the expansion of immunosuppressive cell types.

## LPS-induced late phase of glycolytic reprogramming

While the early phase of glycolytic reprogramming is vital to swiftly rewire the metabolic circuits to fuel the immediate bioenergetic needs of inflammatory machineries (0-4hr), the late phase of glycolytic reprogramming is critical for reconfiguring intracellular metabolism to sustain those needs for extended periods of time (6-24hr), and ultimately for the induction of resolution of inflammation (end phase; 48-72hr) (Curtis et al., 2020). Unlike the early phase where the induction is heavily dependent on post-translational modifications, such as phosphorylation signaling cascades, the late stage of glycolytic reprogramming relies on transcriptional regulation, with HIF-1α and NRF2-mediated transcription being the most important in myeloid cells.

As described previously, HIF-1α can be stabilized by TCA cycle metabolites that mediate the inhibition of PHD activity. Indeed, the reconfiguration of TCA cycle during the late stage of glycolytic reprogramming significantly alters the abundance of TCA cycle metabolites in LPS-activated Mφs. Specifically, this is achieved by two metabolic breakpoints in the TCA cycle. The first breakpoint takes place at the isocitrate dehydrogenase (IDH) level due to the suppression of its mRNA expression (Tannahill et al., 2013; Jha et al., 2015), NO-mediated cysteine nitrosation of IDH (Bailey et al., 2019) and autocrine type I interferon signaling (De Souza et al., 2019). As a result of the suppressed IDH activity, citrate then accumulates and is re-directed towards itaconate through *cis*-Aconitate by *cis*-aconitate decarboxylase or exported into the cytoplasm by citrate transport protein. The accumulation of cytosolic citrate can then be converted into acetyl-CoA through ATP citrate synthase, which is critical for supporting *de novo* lipid synthesis (Ryan and O'Neill, 2020) and histone acetylation of inflammatory genes (Lauterbach et al., 2019; Ting et al., 2024b).

On the other hand, the accumulation of itaconate contributes to the second metabolic breakpoint due to its effect on inhibiting the activity of succinate dehydrogenase (SDH) (Cordes et al., 2016; Lampropoulou et al., 2016), although the inhibitory effect of NO on SDH has also been reported (Jha et al., 2015). Together, with the increased influx of succinate derived from glutamine at the level of α-ketoglutarate (anaplerosis) (Tannahill et al., 2013), both pathways significantly increase the abundance of succinate, which inhibits the activity of PHDs and stabilizes HIF-1α. The stabilized HIF-1α then binds to HIF-1β and the HIF-1 complex activates downstream targeted gene expression, including genes involved in glycolysis and inflammation, such as *Il1b* (Tannahill et al., 2013). Apart from stabilizing HIF-1α, dysregulated succinate metabolism can lead to lysine succinylation, a novel post-translational modification that can regulate enzymes involved in remodeling the epigenome (Park et al., 2013; Tannahill et al., 2013). Specifically, succinylation can induce a 100-Da change in mass of targeted proteins and shield the positively charged lysine side chains (Park et al., 2013). Enzymes, such as PKM2, can be hypersuccinylated on lysine 311 which promotes its tetramer-to-dimer transition, thereby inhibiting its pyruvate kinase activity but activates its role as a co-factor for HIF-1α-dependent transcription (Palsson-McDermott et al., 2015; Wang et al., 2017).

**Figure 3:**
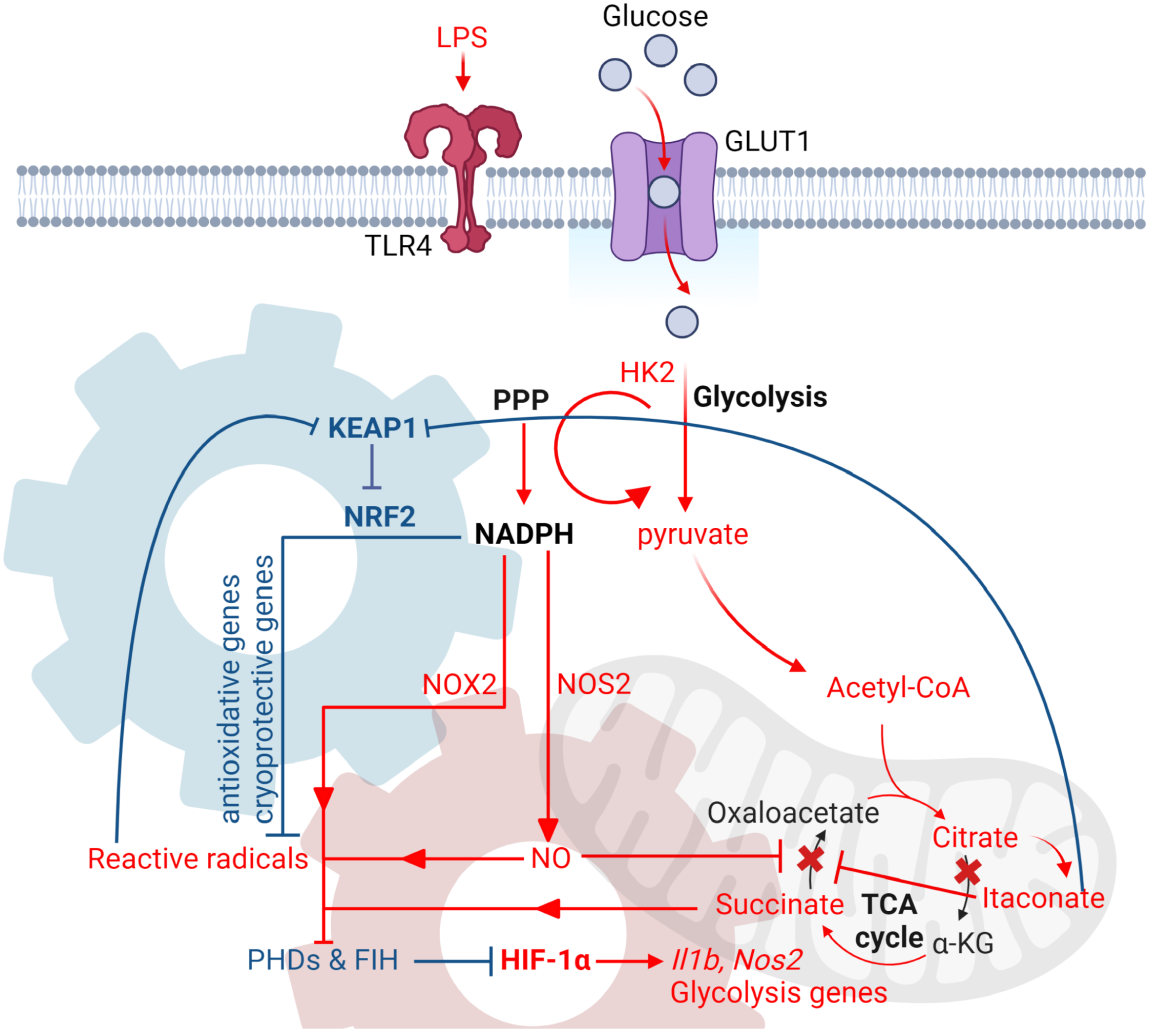
LPS-induced glycolytic reprogramming in myeloid cells. Diagram that illustrates the key events during the late phase of glycolytic reprogramming in myeloid cells upon LPS stimulation. During the late phase, the increased rate of glycolysis then feeds into the TCA cycle, where two metabolic breakpoints take place. The first break happens at the level of isocitrate dehydrogenase (IDH), leading to the accumulation of citrate and itaconate. Citrate can then be converted back to acetyl-CoA by ACLY and supports *de novo* lipid synthesis and production of pro-inflammatory cytokines, as well as histone acetylation of inflammatory genes. The second break happens at the level of succinate dehydrogenase due itaconate-mediated inhibition, thus leading to the accumulation of succinate. Succinate, along with the production of reactive radicals derived from NADPH-dependent inflammatory enzymes, such as NOX2 and NOS2, inhibit the activities of PHD and FIH. This subsequently leads to the activation of HIF-1 and transcription of its targeted inflammatory and glycolytic genes. To limit HIF-1-dependent-inflammation, NRF2 is activated in response to oxidative stress and itaconate-mediated alkylation of KEAP1. The enhanced transcription and translation of NRF2-targeted antioxidative proteins then detoxify ROS by consuming the same pool of NADPH that is also required to stabilize HIF-1α. This in turn increases the activity of PHD and FIH, which leads to the reduction of HIF-1 transcription function and ultimately the fine-tuning of Mφ inflammatory response. Red gear denotes HIF-1α transcriptional circuit, while blue gear denotes NRF2 transcriptional circuit. Both circuits consume NADPH as a mechanism of co-regulation. All figures are made by Biorender.com.

Apart from HIF-1α-dependent transcription, NRF2-mediated transcriptional regulation is also critical for regulating the late-phase of glycolytic reprogramming. As previously described, the accumulation of itaconate due to the remodeling of TCA cycle leads to the alkylation of KEAP1 and subsequent activation of NRF2 (Mills et al., 2018). In addition to mediating the transcription of antioxidative genes, NRF2 has been shown to limit LPS-induced inflammatory responses through inhibiting RNA Polymerase II binding to the promoters of *Il1b* and *Il6* (Kobayashi et al., 2016), suppressing HIF-1α-mediated glycolytic reprogramming (Mills et al., 2018) and suppression of Type 1 IFN responses by decreasing the mRNA stability of STING (Olagnier et al., 2018). More recently, it has also been demonstrated that NRF2 can regulate HIF-1α stability and its transcriptional function in a NADPH-dependent manner (Ting et al., 2023b; Ting et al., 2023a; Ting et al., 2024a). Specifically, it has been shown that the enhanced activation of NRF2 led to the increased transcription and translation of NRF2-targeted apoenzymes (Ting et al., 2023b). These pools are proteins then detoxify ROS by consuming the same NADPH pools that is required to synthesize ROS and stabilize HIF-1α, eventually limiting HIF-1-dependent inflammation and fine-tune Mφ inflammatory responses (**Figure 3**).

Apart from fueling the effector functions of activated Mφs in a sustained manner, the end phase of glycolytic reprogramming is also important for the resolution of inflammation (48-72hr), which is characterized by the subsidence, rather than accumulation, of itaconate and succinate. For instance, a recent study has shown that after the transient accumulation of itaconate and succinate (as a result of the two breakpoints in the TCA cycle), pyruvate dehydrogenase complex and oxoglutarate dehydrogenase complex were inhibited due to the alteration of their lipoylation states (Seim et al., 2019). The inhibition of these complexes led to the normalization of citrate, itaconate and succinate levels, and correlated with a decline of HIF-1α protein levels. Taken together, these studies illustrate that the re-configuration of the TCA cycle is a highly dynamic and time dependent process.

## The role of HIF-1α in bacterial infection models

HIF-1 is a master transcription factor that regulates the expression of hypoxic, inflammatory, and glycolytic genes, thus its role in mediating the inflammatory response of Mφs under hypoxic environment has been previously investigated. Indeed, wound and necrotic tissue foci have less oxygen availability (<1% oxygen) than normal healthy tissue (2.5 – 9% oxygen) (Arnold et al., 1987; Vogelberg and König, 1993; Negus et al., 1997), thus suggesting the possibility that the antimicrobial functions performed by Mφs, such as an increased ability to phagocytose, are regulated in part by the hypoxic environment (Anand et al., 2007). In 2008, Michael Karin’s group was the first group that characterized the relationship between hypoxia and HIF-1α-mediated immunity, where they have shown that NF-кB could bind to the promoter of *Hif1a* in LPS-stimulated Mφs (Rius et al., 2008). They then showed that HIF-1α accumulation in Mφs infected with Group A *Streptococcus* (GAS) and P. *aeruginosa* were dependent on IKK-β, one of the IкB kinases that activates NF-кB by phosphorylating the inhibitor of NF-кB (Häcker and Karin, 2006). Notably, these findings were reproduced in hypoxic conditions where IKK-β could regulate hypoxia-induced HIF-1α expression and activity. Taken together, these results have demonstrated that NF-кB is a hypoxia-regulated transcription factor that activates the transcription of *Hif1a*, providing the first evidence that illustrates how the inflammatory and hypoxic responses of Mφs are integrated.

With respect to how HIF-1α mediates bactericidal activities in Mφs, this was first characterized by Randall Johnson’s group back in 2003, where the group has found that Group B *Streptococcus* (GBS) was more viable in HIF-1α-deficient Mφs compared to its wild-type counterparts, which suggests that HIF-1α is important for intracellular killing of bacterial pathogens (Cramer et al., 2003). This was subsequently confirmed in another study performed by the same group, in which they found that HIF-1α-deficiency in Mφs impaired their intracellular killing ability of both GAS and *P. aeruginosa* (Peyssonnaux et al., 2005). On the other hand, the constitutive activation of HIF-1α in VHL-null mice have in turn increased intracellular killing of GAS and *P. aeruginosa.* To assess the role of HIF-1α in regulating myeloid cell bactericidal function *in vivo*, the group then adopted an infection model of GAS and found that mice with HIF-1α genetic deficiency in myeloid cells developed greater necrotic skin lesions. Mechanistically, the group then discovered that HIF-1α is critical for neutrophil-mediated production of granule proteases and antimicrobial peptides, as well as Mφ-mediated secretion of nitric oxide and TNF-α production. Similar findings were also reported by Braverman et al., where the authors have adopted a murine *M. tuberculosis* model and found that HIF-1α is responsible to regulate many IFN-γ-inducible responses in infected Mφs, such as production of nitric oxide and other inflammatory cytokines and chemokines (Braverman et al., 2016). Several years later, Knight et al. took a step further and demonstrated that HIF-1α-dependent transcription of *Hig2* is critical for driving the formation of lipid droplets in lungs of mice infected with *M. tuberculosis*, an activated immune response from Mφs (Knight et al., 2018). The importance of HIF-1α-mediated antimicrobial activity against M. tuberculosis was also recently shown in human macrophages (Zenk et al., 2021). Finally, the direct binding of HIF-1α to other targeted genes, such as *Il1b*, has also been shown to be critical in limiting *M. marium* infection of zebrafish (Elks et al., 2013; Ogryzko et al., 2019). Specifically, studies have found that *M. marinum* infection of zebrafish induced HIF-1α-dependent transcription of *Il1b*, which then activated neutrophil-mediated production of nitric oxide and protection against infection. Similar to *M. marinum*, mice with genetic deficiency of HIF-1α in their myeloid cells also resulted in increased bacterial burden and necrotic granulomas upon infection by *M. avium* (Cardoso et al., 2015).

Apart from regulating inflammatory responses, HIF-1α is also a central regulator of glycolytic responses, in which its induction serves as the metabolic basis of host immunity. In 2011, Mihai Netea and colleagues proposed the concept of “Trained Immunity” to describe the phenomenon that innate immune cells, such as monocytes and Mφs, can also display immunological memory of past insults (Netea et al., 2011). For instance, Netea’s group first demonstrated that β-glucan derived from *C. albicans* could induce trained immunity in monocytes, where the first stimulation primed their enhanced production of pro-inflammatory cytokines upon the second stimulation (Quintin et al., 2012). Next, Netea’s group then demonstrated that mTOR/HIF-1α-mediated glycolysis metabolically supports the basis of trained immunity (Cheng et al., 2014). Specifically, the group has found that AKT/mTOR/HIF-1α pathways was downstream of β–glucan stimulation, and that training monocytes with β–glucan against *S. aureus* sepsis was impaired in mice with genetic deficiency of HIF-1α in myeloid cells. Apart from fungi, the importance of HIF-1α-mediated glycolytic response in Mφs was also observed in bacterial infection models, such as *M. tuberculosis* (Braverman et al., 2016) and *L. monocytogenes* (Li et al., 2018). Taken together, these studies have collectively demonstrated that HIF-1α-mediated transcription of inflammatory and glycolytic genes underlie Mφ innate immunity against many different bacteria species.

## The role of NRF2 in bacterial infection models

Apart from HIF-1α, NRF2 also plays a central role in regulating Mφ inflammatory responses against bacterial infections, particularly its role in regulating the transcription of its targeted genes. Among all, its transcription of macrophage receptor with collagenous structure (MARCO), an important receptor required for the phagocytosis of bacteria, has been repeatedly shown to be critical in enhancing antibacterial defenses of Mφs. For instance, Harvey et al. have first demonstrated that sulforaphane (SFN)-induced NRF2 activation restored bacteria recognition and phagocytic ability of alveolar Mφs derived from patients with chronic obstructive pulmonary disease (Harvey et al., 2011). Specifically, the authors found that SFN-induced activation of NRF2 significantly enhanced the expression of MARCO and the ability of Mφs to phagocytose *P. aeruginosa*, while the disruption of MARCO or NRF2 impaired their phagocytic abilities. Similar findings were also reported by Pang et al., where the authors found that Early growth response 1 (ERG1)-mediated suppression of phagocytosing *P. aeruginosa* was due to inhibition of NRF2 signaling (Pang et al., 2022). In this study, the authors have found that *P. aeruginosa* induced ERG1 expression and autophagy-related processes during infection of Mφs. The induction of autophagy subsequently enhanced the degradation of p62 and suppressed NRF2 levels. The impaired NRF2 levels then resulted in the reduced transcription of MARCO and Macrophage scavenger receptor 1 (MSR1), which is another scavenger receptor important for phagocytosing bacteria. In addition to *P. aeruginosa*, Wang et al. have also discovered similar results with *E. coli* infection, where they found that IR-61, an inhibitor that disrupts the interaction between KEAP1 and NRF2, augmented Mφs ability to phagocytose bacteria. Mechanically, the authors utilized an *in silico* molecular ligand docking analysis and found that IR-61 bound to KEAP1 and induced NRF2 release and activation (Wang et al., 2023). Finally, Luo et al. have discovered that itaconate is critical in supporting Mφ phagocytic abilities by enhancing NRF2-dependent transcription of *Cd36*, which is another scavenger receptor of Mφs that is involved in internalizing bacteria (Wang et al., 2023). Taken together, these studies have collectively demonstrated that NRF2-dependent transcription of scavenger receptors, such as MARCO, MSR1 and CD36, are critical players in orchestrating Mφ bactericidal responses.

Apart from supporting phagocytosis, NRF2 also regulates Mφ inflammatory responses against bacterial infection through maintaining redox homeostasis, cell survival, the formation of phagolysosomes and iron homeostasis. For instance, Sun et al. showed that activation of NRF2 by oltipraz (OTZ), a synthetic dithiolethione, is critical to protect *M. tuberculosis*-induced oxidative injury and cell death of human Mφs (Sun et al., 2020). Specifically, OTZ-induced NRF2 activation led to the transcription of antioxidative genes and offered cryoprotection against *M. tuberculosis*-induced oxidative stress. Apart from this, Nakajima et al. have found that SFN-induced NRF2 activation increased Mφs control of *M. avium* by promoting the formation and phagolysosome fusion and granuloma formation (Nakajima et al., 2021). This is due to NRF2-dependent transcription of *Slc11a1* (NRAMP1) and *Hmox1* (HO-1) as they are involved in promoting phagosome-lysosome fusion and granuloma formation respectively. Finally, Nairz et al. have shown that nitric oxide production in Mφs is critical for NRF2-dependent transcription of ferroportin-1 (*Fpn1*), an iron exporter that exports iron extracellularly and prevents its acquisition by *S. typhimurium* (Nairz et al., 2013). Specifically, in this study, the authors discovered that Mφs with *Nos2* genetic deficiency have increased intracellular iron storage due to impaired *Fpn1* expression, thus allowing *S. typhimurium* to utilize its iron content. Notably, nitric oxide promoted NRF2 activation and its transcription of *Fpn1*, thus limiting the growth of S*. typhimurium.* Overall, these studies have shown that NRF2 plays a positive role in regulating Mφs responses against bacterial infection by regulating the transcription of its targeted genes that are involved in a variety of cellular processes.

## Conclusion

Recent advances in the immunometabolism field have comprehensively demonstrated how the rewiring of intracellular metabolic circuits are linked to the effector functions of Mφs, such as the transcription of inflammatory cytokines and glycolytic genes. Notably, the transcriptional regulation mediated by HIF-1α and NRF2 play significant roles behind these processes. Although these transcription factors were discovered back in the 1990s and their pathways have been since well characterized, the birth of the immunometabolism field has now revealed novel functions that they can play, as well as the possibility that they can co-regulate each other as a mechanism to fine tune Mφs inflammatory responses. Understanding that multiple transcription factors are responsible for relaying complex information of extracellular changes into host cell responses through the activation or inhibition of transcriptional circuits, future research should be warranted to further elucidate the co-regulation between other transcriptional circuits and identify their underlying co-regulators.

HIF-1α-mediated transcriptional responses in Mφs is a general phenomenon against many types of bacteria (Werth et al., 2010). Notably, the transcriptional regulation of HIF-1α on inflammatory and glycolytic genes primarily orchestrate Mφ inflammatory responses, and the oxygen availability (degree of hypoxia) in the environment helps to fine-tune its transcriptional output and the extent of inflammation. While this multi-layered regulation of HIF-1α responses is critical for Mφ immunity and has been well characterized, it also opens the possibility that pathogens can evolve and adapt to this for its survival. Indeed, a recent study has shown that hypoxia-induced HIF-1α-response in Mφs limited the replication of *C. burnetiid* without affecting its viability, thus allowing it to persist chronically and this may be linked to the development of chronic Q fever (Hayek et al., 2019). Future research should thus be warranted to investigate how pathogens have developed mechanisms to resist and adapt to HIF-1α-mediated immunity in Mφs, particularly in the context of chronic bacterial infection or re-infection models.

Similar to HIF-1α, NRF2 also plays an essential role in mediating Mφ responses against bacterial infection, primarily by enhancing its phagocytic abilities through upregulating its targeted scavenger reports, as well as maintaining a favorable redox environment and cell survival. Although many studies have supported a positive role of NRF2 in promoting Mφ bactericidal responses, an interesting study has recently shown that *M. tuberculosis*-induced early NRF2-responses in alveolar Mφs in fact hindered their ability to elicit a robust inflammatory response and its effective control of bacterial growth (Rothchild et al., 2019). This suggests that *M. tuberculosis* may have developed mechanisms over evolutionary time to infect a certain population of Mφs that preferentially upregulate antioxidative defense mechanisms over classical inflammatory responses. This may also explain the reason behind the conflicting effectiveness of pharmacological NRF2 activators in limiting bacterial infection, especially when compared against different types of bacteria species (Ali et al., 2020). Therefore, future research should be warranted to elucidate the mechanisms behind how different types of bacteria adapt to NRF2-mediated responses in Mφs.

## Author contributions

KT: Funding acquisition, Visualization, Writing – original draft, Writing – review & editing.
